# Sulfonamide Allergies

**DOI:** 10.3390/pharmacy7030132

**Published:** 2019-09-11

**Authors:** Amber Giles, Jaime Foushee, Evan Lantz, Giuseppe Gumina

**Affiliations:** 1 Presbyterian College School of Pharmacy, 307 N. Broad St., Clinton, SC 29325, USAggumina@presby.edu (G.G.); 2 Janssen Scientific Affairs, LLC., 1125 Trenton Harbourton Rd., Titusville, NJ 08560, USA; 3 Edward Via College of Osteopathic Medicine Carolinas Campus, 350 Howard St., Spartanburg, SC 29303, USA; 4 Spartanburg Regional Healthcare System, 101 E. Wood St., Spartanburg, SC 29303, USA; elantz@srhs.com

**Keywords:** antimicrobials, allergy, hypersensitivity, cross-reaction, sulfonamide, sulfa

## Abstract

As one of the earliest developed antimicrobial classes, sulfonamides remain important therapeutic options for the empiric and definitive treatment of various infectious diseases. In the general population, approximately 3–8% of patients are reported to experience a sulfonamide allergy. Sulfonamide allergies can result in various physical manifestations; however, rash is reported as the most frequently observed. In patients with human immunodeficiency virus (HIV), dermatologic reactions to sulfonamide antimicrobial agents occur 10 to 20 times more frequently compared to immunocompetent patients. This article describes the incidence, manifestations, and risk factors associated with sulfonamide allergies. The potential for cross-reactivity of allergies to sulfonamide antimicrobials with nonantimicrobial sulfonamide medications is also reviewed. Data suggest that substitutions at the N_1_ and N_4_ positions are the primary determinants of drug allergy instead of the common sulfonamide moiety. For patients with an indication for a sulfonamide antimicrobial with a listed allergy, it is important for healthcare practitioners to adequately assess the allergic reaction to determine appropriate management. Rechallenge and desensitization strategies may be appropriate for patients with delayed maculopapular eruptions, while alternative treatment options may be prudent for more severe reactions. Available data suggests a low risk of cross-allergenicity between sulfonamide antimicrobial and nonantimicrobial agents.

## 1. Introduction

As one of the earliest developed antimicrobial classes, sulfonamides have proven utility for a variety of infectious diseases. For many indications, sulfonamides have been replaced by safer, and in some cases, more efficacious alternatives. Despite the relatively high incidence of adverse effects, sulfonamides maintain their place as treatment of choice for certain infectious diseases including *Pneumocystis jirovecii* pneumonia (PCP), uncomplicated cystitis, and *Stenotrophomonas maltophilia*. In the general population, approximately 3–8% of patients are reported to experience a sulfonamide allergy [1,2,3,4,5,6,7,8]. Sulfonamide allergies can result in various physical manifestations; however, rash is reported as the most frequently observed reaction to sulfonamide antimicrobials. Skin eruptions may occur in 1.5–3% of patients who are immunocompetent and in rates as high as 30% in patients with human immunodeficiency virus (HIV) [1]. More serious reactions, including Stevens–Johnson Syndrome (SJS), occur with lower frequencies but may lead to significant morbidity and mortality. This article describes the incidence, manifestations, and risk factors associated with sulfonamide allergies. The potential for cross-reactivity of allergies to sulfonamide antimicrobials with nonantimicrobial sulfonamide medications is also reviewed.

## 2. First-Line Indications

Trimethoprim–sulfamethoxazole (TMP–SMX) is the most commonly utilized sulfonamide antimicrobial and is useful for treatment of a variety of infectious diseases. The most recent guidelines published by the Infectious Diseases Society of America (IDSA) recommend TMP–SMX as one of the first-line treatment options for uncomplicated urinary tract infections (UTIs) when local *Escherichia coli* resistance rates are below 20 percent or when the identified bacteria strain is susceptible to the agent [9]. Favorable data evaluating clinical and microbiological cure rates in patients with uncomplicated UTIs have been published utilizing a dose of 160/800 mg twice daily [10,11,12,13]. The guideline recommends a duration of therapy of 3 days for uncomplicated cystitis [9]. Other first line treatment options are available for patients with sulfonamide antimicrobial allergies diagnosed with uncomplicated cystitis, including nitrofurantoin and fosfomycin [9].

TMP–SMX is also recommended by the IDSA as a first-line empiric treatment option for moderate, purulent skin and soft tissue infections (SSTIs), in addition to incision and drainage. It can also be used as definitive treatment for moderate SSTIs caused by methicillin-resistant *Staphylococcus aureus* (MRSA) [14]. These infections can be treated with a dose of 80/400 mg or 160/800 mg by mouth twice daily for a treatment duration of 5 to 10 days. An alternative empiric option for sulfonamide-intolerant adult patients with moderate SSTIs is doxycycline [14].

*Stenotrophomonas maltophilia* is a Gram-negative bacillus that is found in water and soil. This organism is commonly associated with nosocomial infections such as hospital-acquired and ventilator-associated pneumonia. The organism has also been associated with other sources of infection; however, the significance is questionable. TMP–SMX remains the drug of choice for treating infections caused by *S. maltophilia*. In patients who cannot tolerate or organisms not susceptible to TMP–SMX, fluoroquinolones are an alternative treatment option with similar clinical success [15].

*Pneumocystis jirovecii* is a ubiquitous fungal organism [16]. Initial infection most often occurs in childhood; two-thirds of healthy children have antibodies to *P. jirovecii* by age 2–4 years [17]. Clinically relevant disease, Pneumocystis pneumonia (PCP), is often due to reactivation or new infection in immunosuppressed patients. In patients with severe immunosuppression, the rate of mortality ranges from 20% to 40% of patients who receive treatment [16]. According to current guidelines, the drug of choice for PCP prophylaxis in patients with HIV, solid organ transplantation (SOT), hematological malignancies, solid tumors, and allogeneic stem cell transplantation is TMP–SMX [16,18,19,20,21]. Alternative prophylaxis options include dapsone, aerosolized pentamidine administered via the Respirgard II^®^ nebulizer, atovaquone, and atovaquone plus pyrimethamine plus leucovorin [16]. Oral clindamycin plus primaquine, intermittent pentamidine, and aerosolized pentamidine administered by non-Respirgard II^®^ nebulizers are not recommended alternatives in the event of a sulfonamide allergy due to lack of efficacy data. Often in combination with corticosteroids, TMP–SMX is also the drug of choice for treatment of PCP at high doses (15–20 mg/kg/day divided every 6–8 h) due to superior efficacy, better tolerability, and/or ease of administration as compared to other treatment options such as atovaquone, dapsone plus trimethoprim, and clindamycin plus primaquine [16].

The protozoan *Toxoplasma gondii* is an opportunistic organism that can cause a focal encephalitis in patients who are immunosuppressed [16]. *T. gondii* is more prevalent in certain geographic regions such as Europe, Africa, and Latin America but is also seen in the United States. Like PCP, many cases are due to reactivation of latent disease in the immunocompromised but may be caused by primary infection. In addition, the drug of choice for primary and secondary prophylaxis is also TMP–SMX. Alternative options for prophylaxis include dapsone with pyrimethamine/leucovorin or atovaquone with or without pyrimethamine/leucovorin. The preferred treatment regimen for *Toxoplasma* encephalitis (TE) includes another sulfonamide antimicrobial. Weight-based sulfadiazine is used in combination with pyrimethamine and leucovorin to treat patients diagnosed with disease. A commonly utilized alternative treatment is TMP–SMX (5 mg/kg every 12 h). Non-sulfonamide options for treatment of TE include pyrimethamine/leucovorin plus clindamycin or atovaquone with or without pyrimethamine/leucovorin [16].

While alternative options exist for the prevention and treatment of the aforementioned infectious diseases, it is important to remember that many of these alternative therapies may have either decreased efficacy or increased toxicity compared to sulfonamide-based ones. Therefore, it is crucial to screen patients accurately for true sulfonamide allergies versus intolerances when choosing antimicrobial therapy.

## 3. Sulfonamide Allergy

### 3.1. Mechanisms and Manifestations of Sulfonamide Allergy

**Figure 1:**
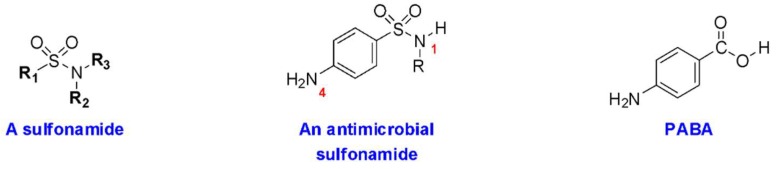
Structures of the sulfonamide functional group, an antimicrobial sulfonamide, and structural similarity to *p*-aminobenzoic acid (PABA).

**Figure 2:**
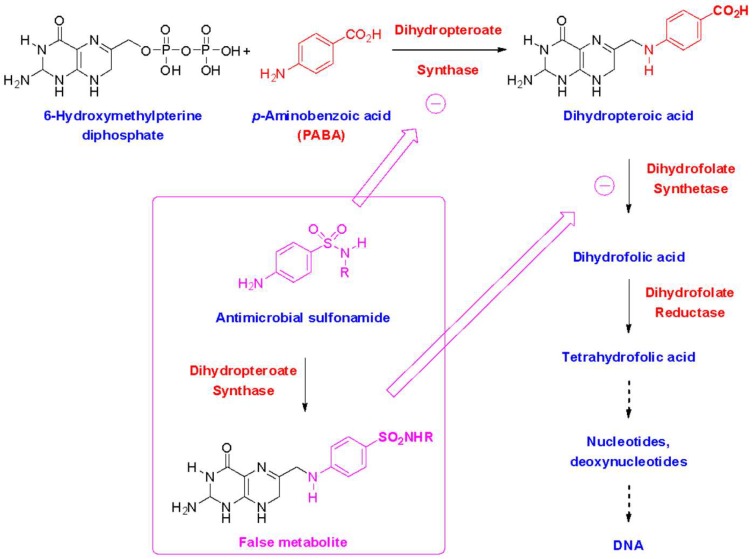
Mechanisms of action of antimicrobial sulfonamides.

**Figure 3:**
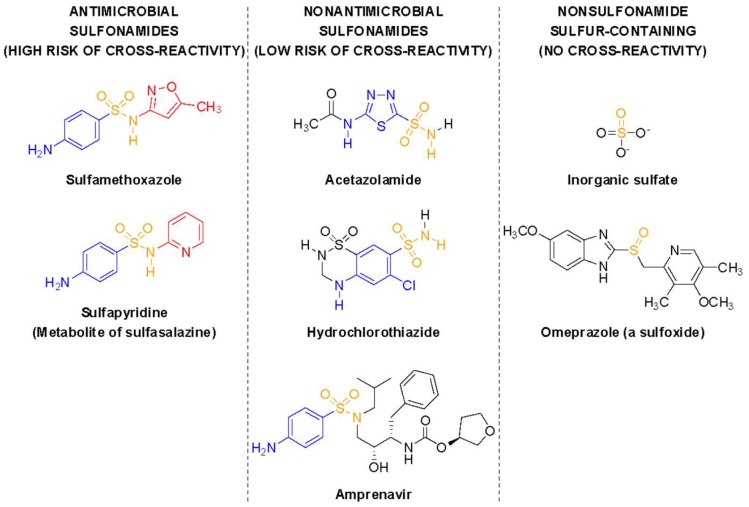
Examples of sulfonamide antimicrobials, sulfonamide nonantimicrobials, and nonsulfonamide sulfur-containing compounds. In color are the three structural elements (sulfonamide in orange, N_4_-arylamine in blue, N_1_-heterocycle in red) required for potent antimicrobial activity and associated with typical sulfa allergy.

Sulfonamide medications are often divided into two subsets—sulfonamide antimicrobials and sulfonamide nonantimicrobials (Table 1) [2]. All sulfonamides contain an NH_2_-SO_2_ moiety; however, sulfonamide antimicrobials also contain an aryl-amine (-Ar-NH_2_) at the N_4_ position and a five- or six-membered, nitrogen-containing ring at the N_1_ position (Figure 1). The arylamine moiety is responsible for the mechanism of action of sulfonamide antimicrobials, due to the similarity between the 4-aminobenzenesulfonamide and *p*-aminobenzoic acid (PABA), required for microbial synthesis of dihydrofolic acid [22]. This similarity provides a dual mechanism of action: Competitive inhibition of microbial dihydropteroate synthetase, and incorporation of the sulfonamide in place of PABA into a false metabolic intermediate that cannot be converted to dihydrofolate by dihydrofolate synthetase (Figure 2). The N_1_-heterocyclic ring increases the acidity of the N_1_ proton, thus allowing to better mimic the acidic proton of PABA. The increased acidity also greatly improves the water solubility of sulfonamide antimicrobials, which is important since the undissociated forms of these molecules and their acetate metabolites tend to have low solubility, which can be responsible for crystalluria. These additional groups are believed to be the primary determinant of allergy, rather than the base NH_2_-SO_2_ moiety contained in all sulfonamides (Figure 3) [1,3,23,24,25]. The majority of nonantimicrobial sulfonamides lack these additional groups, with the exception of the antiretroviral agents, amprenavir and fosamprenavir, which contain an N_4_-arylamine group (but not an N_1_-heterocyclic ring) [2].

**Figure 4:**
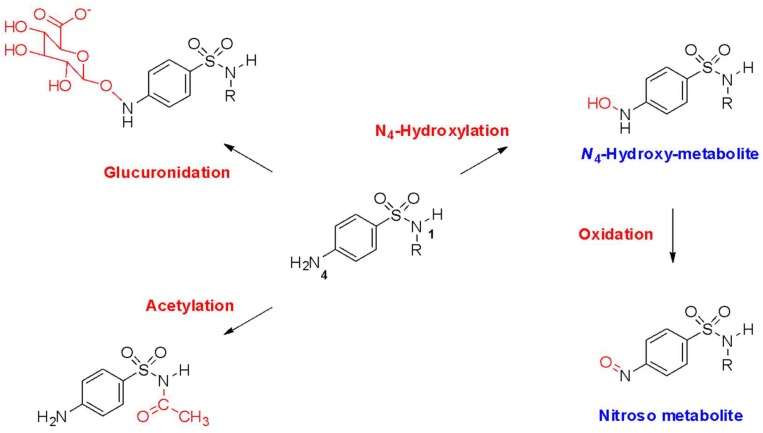
Metabolism of antimicrobial sulfonamides.

Patients experiencing a sulfonamide antimicrobial allergy may experience a variety of clinical manifestations. These may include hypersensitivity reactions from each of the Gel and Coombs classifications. Type 1 immunoglubulin E (IgE)-mediated reactions result in manifestations such as anaphylaxis, angioedema, and urticaria [2]. Regarding IgE-mediated type 1 reactions, it is important to note that the sulfonamide-defining NH_2_-SO_2_ moiety has not been found to be bound by IgE. Instead, the N_1_ heterocyclic ring has been found to be recognized by IgE, especially if a methyl group is in the β position on the isoxazole ring [1,23]. Non-type 1 reactions are mediated by three potential mechanisms: (1) the parent molecule or reactive metabolites acting as haptens; (2) the molecule binding to a native protein stimulating a cellular or humoral immune response, or (3) a cellular protein causing direct cytotoxicity, or stimulation of T-cells to produce an immune response [1]. The non-type-1 hypersensitivity reactions are typically associated with metabolites of the sulfonamide antimicrobial agents. Sulfonamide antimicrobial agents undergo acetylation, glucuronidation, and hydroxylation to various metabolites. A particular metabolite associated with allergic immunogenicity is the N_4_-hydroxylated metabolite, which can then be oxidized to a reactive nitroso compound (Figure 4). This compound can be reduced through a reaction utilizing glutathione, or acetylated using the NAT 2 enzyme. Patients who are slow-acetylator phenotypes or who have deficiencies in glutathione may be predisposed to experience non-type-I hypersensitivity reactions due to decreased ability to metabolize these sulfonamide antimicrobial metabolites. The reactive nitroso compound can bind directly to T cells to illicit maculopapular eruptions, including SJS [2].

The most common manifestation of a true sulfonamide antimicrobial reaction is a maculopapular eruption. This rash, which may occur in conjunction with a fever, typically presents 1–2 weeks following the introduction of SMX therapy and often dissipates over a similar time course, within 1–2 weeks of withdrawal of the sulfonamide antimicrobial [4]. However, this dermatologic toxicity does not require absolute discontinuation of the sulfonamide antimicrobial and in fact, many patients can continue treatment with the sulfonamide antimicrobial without cessation of therapy. Protocols for reintroduction have been developed for HIV-positive and HIV-negative patients that develop a delayed maculopapular eruption after TMP–SMX administration [26,27,28,29,30]. Bonfanti et al. performed a randomized trial of TMP–SMX desensitization versus re-challenge (single dose) and found equivalent success rates with both approaches (79.5% vs. 72%, respectively) [31].

While most maculopapular eruptions will resolve within several days, the presence of blistering, involvement of mucous membranes, and development of arthralgias may be signs of SJS. When the body surface area affected exceeds 30%, toxic epidermal necrolysis (TEN) is diagnosed. Patients with SJS and TEN require hospitalization, often in intensive care and/or specialized burn units [4]. Reintroduction of a sulfonamide antimicrobial should generally be reserved for patients that develop a delayed maculopapular rash; however, Douglas et al. describe two patient cases of successful TMP–SMX desensitization with a history of SJS [32]. While successful in these cases, reintroduction of a sulfonamide antimicrobial in a patient with a history of SJS remains unadvisable.

The predominance of self-reported adverse drug reactions to sulfonamide antimicrobials are comprised of gastrointestinal (GI) upset and dermatologic reactions [8]. As GI side effects are characterized as medication intolerances, it is important to differentiate medication intolerances from allergic reactions.

### 3.2. Incidence and Risk Factors

Determining the true incidence of sulfonamide allergies is challenging, as allergy history is often self-reported by patients. A retrospective review of electronic medical records from patients receiving care in San Diego County through Kaiser Permanente aimed to determine the incidence and prevalence of self-reported antimicrobial allergies [5]. The study evaluated 411,543 patients who had at least one outpatient visit during 2007. Antimicrobial allergy rates were calculated by determining the number of patients reported as having an allergy to the antimicrobial they were prescribed in 2007 divided by the sum of all patients who received that antimicrobial during 2007. The antimicrobial allergy rates were then stratified by gender to account for differences in incidence reporting. Sulfa allergy incidence among males and females was found to be 2.23 percent (1.91–2.59) and 3.42 percent (3.13–3.74), respectively. Additionally, sulfa antimicrobials were associated with the highest incidence rates of antimicrobial allergies for both males and females compared to penicillin, cephalosporin, fluoroquinolone, tetracycline, and macrolide antimicrobials (*p* < 0.0001) [5].

In an inpatient setting, Lee and colleagues reviewed the incidence of self-reported antimicrobial allergies in patients requiring antimicrobial therapies over a two-month time period in an academic medical center [6]. Of the 2013 patients identified as requiring an antimicrobial agent, 138 (7.3%) reported an allergy to sulfonamides. The only allergy reported more frequently was a penicillin allergy (n = 295; 15.6%). Where 85 patients reported multiple antimicrobial agent allergies, the most commonly reported allergy combination was penicillin with a sulfonamide (n = 22; 26%) [6].

In previous publications, 3–8% of patients treated with a sulfonamide antimicrobial report experiencing an allergic reaction [1,2,3,4,5,6,7,8]. The most significant reported risk factor for a sulfonamide allergy is consistently HIV-positivity, specifically in patients with AIDS [33]. In contrast to the 3–8% reaction rate detected in the general population, Carr and colleagues found that hypersensitivity reactions occurred in 27% of patients with HIV that received TMP–SMX for the treatment of PCP. Additionally, adverse reactions to TMP–SMX in this study decreased with HIV progression, possibly attributable to a decrease in T lymphocytes [34].

The ability to prospectively identify patients at higher risk of allergy to sulfonamide antimicrobials would be useful in certain clinical scenarios; however, there are not validated diagnostic tests to his end. Also, there do not appear to be consistent genetic markers that reliably predict sulfonamide drug allergy [24]. Skin testing with non-irritating concentrations of the drug may seem like a favorable approach; however, the predictive utility of an IgE-mediated reaction using skin testing is limited and type 1 allergic reaction to sulfonamide antimicrobials is less common than other types [33]. The American Academy of Allergy, Asthma, and Immunology (AAAAI) recommends against elective skin testing to evaluate for allergy to non-β-lactam antimicrobials; instead, skin testing should be reserved for patients that require, or are anticipated to require, the non-β-lactam antimicrobial. If skin testing is employed, the AAAAI recommends utilizing a non-irritating concentration of the drug: 800 µg/mL (based on the SMX component) of TMP–SMX [33].

### 3.3. Cross-Reactivity of Sulfonamide-Containing Agents

The sulfonamide (SO_2_-NH_2_) moiety is contained in many medications. Agents containing this structural feature can be divided into sulfonamide-containing antimicrobials and sulfonamide-containing nonantimicrobials. The Food and Drug Administration (FDA)-approved antimicrobial sulfonamide agents currently available in the United States include (in order of labeling approval): Sulfadiazine, sulfacetamide, sulfasalazine, sulfanilamide, and sulfamethoxazole [2,35,36,37,38,39]. Sulfacetamide includes a listed precaution in its labeling describing the potential for cross-sensitivity between different sulfonamides. Structural similarities between these antimicrobial agents include a five- to six-membered nitrogen-containing ring attached to the N_1_ nitrogen of the sulfonamide group and an N_4_-arylamine group. The substitutions at these positions are associated with the degree of immunologic response, as they affect the medication’s acetylation, hydroxylation, and glucuronidation to various metabolites that may elicit an allergic reaction [4]. Since all antimicrobial agents share these structural similarities, a high risk of cross-reactivity exists among the sulfonamide antimicrobial agents. Most nonantimicrobial sulfonamides contain neither of these structural features; in fact, none of the nonantimicrobial sulfonamides have an N-containing ring attached to the N_1_ nitrogen of the sulfonamide group, which is required for potent antimicrobial activity [2].

Sulfonamide-containing nonantimicrobial agents (Table 1) include agents from therapeutic classifications such as thiazide and loop diuretics, carbonic anhydrase inhibitors, nonsteroidal anti-inflammatory drugs, sulfonylureas, antiretrovirals, and 5HT-3 receptor agonists [2]. These nonantimicrobial sulfonamides do not undergo metabolism to the N_4_-hydroxylated metabolite associated with SJS and will not bind to IgE at the N_1_ position and, therefore, are unlikely to cause cross-reactivity, even in patients who have experienced type 1 hypersensitivity or serious non-type-1 hypersensitivity reactions to sulfonamide antimicrobial agents. While these agents lack structural features associated with allergy, controversy exists on whether it is safe to administer a sulfonamide-containing nonantimicrobial agent to a patient with a documented hypersensitivity to a sulfonamide-containing antimicrobial agent. Providers may elect a cautious approach by avoiding nonantimicrobial sulfonamides altogether for fear of cross-reactivity; however, this approach may not be necessary based on the tolerability of these agents in patients with reported sulfa allergy [2].

Strom and colleagues published a retrospective cohort study using an electronic database of medical records from outpatient medicine practitioners in the United Kingdom to assess risk factors for sulfonamide cross-reactivity [3]. Patients who received a systemic sulfonamide antimicrobial prescription and subsequently received a sulfonamide nonantimicrobial prescription at least 60 days later than the antimicrobial agents between the years of 1987–1999 were included in the study. The patients were divided into two groups. The study group was comprised of the patients who developed a condition comparable to an allergic reaction within 30 days following receipt of the sulfonamide antimicrobial prescription. The comparison group was comprised of those patients who did not develop an event within 30 days following receipt of the sulfonamide antimicrobial prescription. The primary outcome was development of a presumed allergic reaction within 30 days after the first prescription for a sulfonamide nonantimicrobial. Of patients in the study group who had a prior hypersensitivity reaction after sulfonamide antimicrobial receipt, 96 out of 969 (9.9%) had an allergic reaction within 30 days after receipt of a sulfonamide nonantimicrobial compared to 315 out of 19,257 (1.6%) patients in the comparison group who experienced an allergic reaction within 30 days after receipt of a sulfonamide nonantimicrobial without prior hypersensitivity after sulfonamide antimicrobial receipt. The investigators also compared allergic reaction rates within 30 days of receipt of a penicillin product for patients with and without prior sulfonamide antimicrobial hypersensitivity reactions. A total of 717 of 5115 (14%) of patients with a prior hypersensitivity reaction to a sulfonamide antimicrobial had an allergic reaction within 30 days of receipt of a penicillin compared to 2307 of 112,935 (2%) of patients without a prior hypersensitivity reaction to a sulfonamide antimicrobial developing an allergic reaction within 30 days of receipt of a penicillin. This study concluded an association exists between sulfonamide antimicrobial hypersensitivity and subsequent sulfonamide nonantimicrobial allergic reaction. The results also suggest that this association may be due to patients with sulfonamide allergies having an increased risk for subsequent allergic reactions, including penicillins, rather than a cross-reactivity to sulfonamide nonantimicrobial agents [3].

Hemstreet and colleagues conducted a prospective observational study to characterize sulfonamide allergies in hospitalized adult patients [7]. Of the 94 patients who met inclusion criteria for the study, 42 patients (45%) reported the drug responsible for their sulfonamide allergy as TMP–SMX, and 42 (45%) were unable to recall the medication responsible for their allergy. The majority of patients reported rash or hives (n = 59; 63%) as the clinical manifestation of their allergy, with 13 (14%) reporting anaphylaxis, and two (2%) reporting Stevens–Johnson’s Syndrome. Thirteen of the patients (14%) reporting a sulfonamide allergy cited the reaction as gastrointestinal in nature, where six (6%) could not recall the clinical manifestation resulting from their reported sulfonamide allergy. A second aim of the study was to determine the frequency of cross-allergenicity among patients who report sulfonamide allergies and receive nonantimicrobial sulfonamide agents. Forty of the patients (43%) self-reported sulfonamide nonantimicrobial agent use in the outpatient setting. These medications included furosemide (n = 24; 60%), hydrochlorothiazide (n = 10; 25%), sulfonylureas (n = 7; 18%), celecoxib (n = 6; 15%), sumatriptan (n = 1; 3%), and dapsone (n = 1; 3%). Twenty percent of this subset of patients reported receipt of greater than one sulfonamide nonantimicrobial, and the median duration of use of the sulfonamide nonantimicrobial agent was 6.2 years. No hospitalizations as a result of an allergic reaction had occurred for the patients receiving a nonantimicrobial sulfonamide agent. Further evaluation of patient data showed that nine of the 94 total patients (10%) were prescribed a sulfonamide nonantimicrobial who were not receiving it in the outpatient setting prior to admission. No adverse effects were reported among patients who received a nonantimicrobial sulfonamide, even among patients who reported a life-threatening allergy. This study concluded that many patients with self-reported sulfonamide allergies are often able to receive sulfonamide nonantimicrobial agents with no clinical manifestations of a cross-reactive allergic reaction [7].

Wulf and colleagues reviewed case reports and series examining the risk of cross-reactivity between sulfonamide antimicrobial and nonantimicrobial agents [2]. Over a 20-year time period, they found nine case reports suggesting cross-reactivity between sulfonamide antimicrobial and nonantimicrobial agents. The majority of these reports suggesting an association were with diuretics. In addition to the limited number of reports published over a relatively long time period, the authors highlight the lack of testing done in these case studies to determine true cross-reactivity between agents versus multiple drug allergy syndrome. It is also important to note that none of the nine case reports suggesting cross-allergenicity involved amprenavir or fosamprenavir, the sulfonamide agents with arylamine N_4_ substitutions [2].

Benzocaine, dapsone, acebutolol and procainamide are medications that include an arylamine group that resembles the N_4_ substitution, but lack the sulfonamide moiety [1]. Due to the IgE binding observed at this location, several of these medications have a listed warning for administration in patients with a history of a sulfonamide allergy. There is no available evidence to support or refute a cross-reactivity between these medications and a sulfonamide antimicrobial allergy. However, benzocaine has been associated with numerous case reports of anaphylaxis and the arylamine group could be a determinant of this hypersensitivity reaction [1].

**Figure 5:**
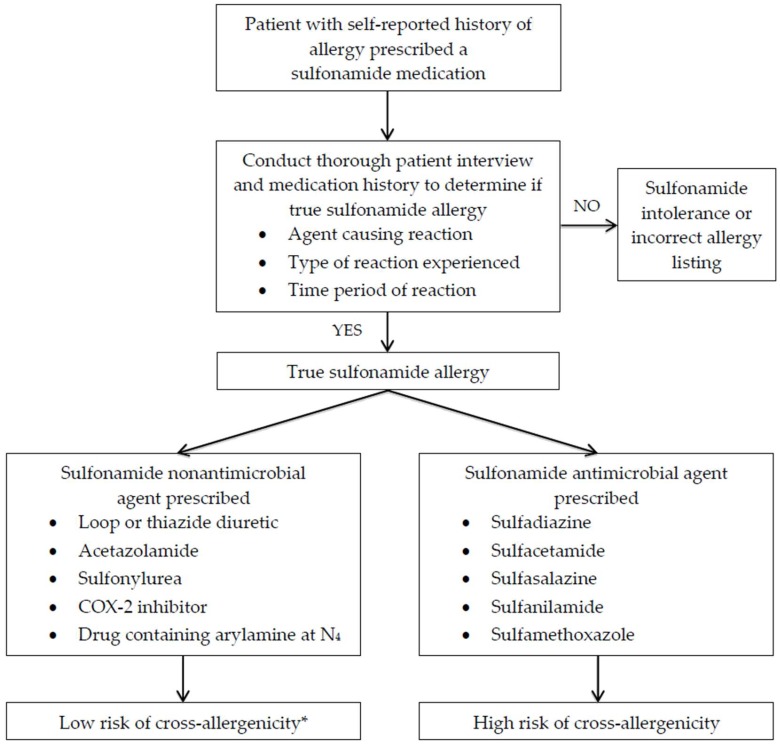
Decision making in patients with sulfonamide antimicrobial allergies. Chart adapted from Ref. [40]. * Patient with true sulfonamide allergy may be at risk for multiple drug allergy syndrome despite low risk of cross-reactivity.

Based on review of the available evidence, sulfonamide antimicrobial agents do not appear to have cross-reactivity with nonantimicrobial sulfonamide agents. The authors would suggest using the schematic in Figure 5 to educate healthcare practitioners on the risk of cross-reactivity among sulfonamide-moiety-containing agents. While the risk of cross-reactivity between sulfonamide antimicrobial and nonantimicrobial agents is low, it is important to note that patients with sulfonamide allergies may have increased risk of multiple drug allergies and may experience allergic reactions to sulfonamide nonantimicrobial agents, but not as a result of cross-reactivity from the sulfonamide moiety.

## 4. Conclusions

Despite decades of therapeutic utilization, sulfonamide antimicrobial agents remain important therapeutic options for the empiric and definitive treatment of various infectious diseases. TMP–SMX is a first-line recommendation in the treatment and/or prevention of PCP, uncomplicated cystitis, moderate purulent SSTI, TE, and *S. maltophilia*. Of the antimicrobial classifications, sulfonamides are associated with some of the highest rates of allergic reactions. Compared to the general population, patients with HIV are at increased risk for experiencing allergic reactions to sulfonamide antimicrobials. While the sulfonamide SO_2_-NH_2_ moiety is also found in several nonantimicrobial medications, substitutions at the N_1_ and N_4_ positions are the primary determinants of drug allergy instead of the common sulfonamide moiety. For patients with an indication for a sulfonamide antimicrobial with a listed allergy, it is important for healthcare practitioners to adequately assess the allergic reaction to determine appropriate management. Rechallenge and desensitization strategies may be appropriate for patients with delayed maculopapular eruptions, while alternative treatment options may be prudent for more severe reactions. Available data suggests a low risk of cross-allergenicity between sulfonamide antimicrobial and nonantimicrobial agents.

## Figures and Tables

**Table 1:** Antimicrobial and nonantimicrobial sulfonamides in the United States [2].

Class	Drugs
Antimicrobials	
Sulfonamides	Sulfacetamide
	Sulfadiazine
	Sulfamerazine
	Sulfamethoxazole
	Sulfanilamide
	Sulfapyridine
	Sulfasalazine
	Sulfathiazole
	Sulfisoxazole
Nonantimicrobials	
Antivirals	Amprenavir *
	Darunavir
	Fosamprenavir *
	Tipranavir
Carbonic anhydrase inhibitors	Acetazolamide
	Brinzolamide
	Dorzolamide
	Methazolamide
COX-2 inhibitors	Celecoxib
	Rofecoxib
	Valdecoxib
Loop diuretics	Bumetanide
	Furosemide
	Torsemide
Sulfonylureas	Acetohexamide
	Chlorpropamide
	Glimepiride
	Glipizide
	Glyburide
	Tolazamide
	Tolbutamide
Thiazide diuretics	Bendroflumethiazide
	Benzthiazide
	Chlorothiazide
	Chlorthalidone
	Cyclothiazide
	Hydrobenzthiazide
	Hydrochlorothiazide
	Methyclothiazide
	Polythiazide
	Quinethazone
Triptans	Almotriptan
	Eletriptan
	Frovatriptan
	Naratriptan
	Rizatriptan
	Sumatriptan
	Zolmitriptan
Miscellaneous	Diazoxide
	Indapamide
	Metolazone
	Probenecid
	Tamsulosin
	Zonisamide

* Contain an N_4_-arylamine group.
